# Management of Ocular Diseases Using Lutein and Zeaxanthin: What Have We Learned from Experimental Animal Studies?

**DOI:** 10.1155/2015/523027

**Published:** 2015-11-05

**Authors:** Chunyan Xue, Richard Rosen, Adrienne Jordan, Dan-Ning Hu

**Affiliations:** ^1^Department of Ophthalmology, Jinling Hospital, School of Medicine, Nanjing University, Nanjing, Jiangsu 210002, China; ^2^Department of Ophthalmology, The New York Eye and Ear Infirmary of Mount Sinai, Ichan School of Medicine at Mount Sinai, New York, NY 10003, USA; ^3^Department of Pathology, The New York Eye and Ear Infirmary of Mount Sinai, Ichan School of Medicine at Mount Sinai, New York, NY 10003, USA

## Abstract

Zeaxanthin and lutein are two carotenoid pigments that concentrated in the retina, especially in the macula. The effects of lutein and zeaxanthin on the prevention and treatment of various eye diseases, including age-related macular degeneration, diabetic retinopathy and cataract, ischemic/hypoxia induced retinopathy, light damage of the retina, retinitis pigmentosa, retinal detachment, and uveitis, have been studied in different experimental animal models. In these animal models, lutein and zeaxanthin have been reported to have beneficial effects in protecting ocular tissues and cells (especially the retinal neurons) against damage caused by different etiological factors. The mechanisms responsible for these effects of lutein and zeaxanthin include prevention of phototoxic damage by absorption of blue light, reduction of oxidative stress through antioxidant activity and free radical scavenging, and their anti-inflammatory and antiangiogenic properties. The results of these experimental animal studies may provide new preventive and therapeutic procedures for clinical management of various vision-threatening diseases.

## 1. Introduction

Zeaxanthin and lutein are two carotenoid pigments that belong to the xanthophylls subclass. They cannot be synthesized in mammals and must be obtained from the diet for distribution to various tissues, especially the retina [1–3]. Zeaxanthin and lutein are most dense at the center of the fovea in the yellowish pigmented area called the macula lutea and are referred to as macular pigment. The macular pigment is tissue protective, acting via antioxidant, anti-inflammatory, and light-screening properties [1–6]. Low systemic and retinal levels of lutein and zeaxanthin are adversely associated with the risk of age-related macular disease (AMD) and diabetic retinopathy [7–10]. Various observational and interventional studies have suggested that the supplementation of lutein and zeaxanthin might reduce the risk of AMD [1, 3, 11–18].

Numerous reports have been published studying the effects of lutein and zeaxanthin on various ocular diseases (AMD, diabetic retinopathy and cataract, ischemic/hypoxia induced retinopathy, light damage of the retina, retinitis pigmentosa, retinal detachment, and uveitis) in experimental animal models [19–51] (Table 1). In this review, we describe the effects of lutein and zeaxanthin and their underlying molecular mechanisms in experimental animal models for various ocular diseases and explore the role of these xanthophylls in the clinical management of vision-threatening diseases.

## 2. Diabetic Retinopathy

Diabetic retinopathy is one of the most common causes of blindness in developed countries. Despite the tighter control of blood glucose and the advances in treatment of diabetic eye diseases, rates of diabetic retinopathy in the United States have increased by 89% over the last decade. Therefore, search for the novel procedures for the prevention and treatment of diabetic retinopathy is urgently required [1].

Zeaxanthin and lutein have been reported to have therapeutic effects in experimental animal models of diabetic retinopathy through their antioxidant and anti-inflammation effects. Injection of streptozotocin (STZ), a compound that is toxic to the insulin-producing beta cells of the pancreas, can be used to produce an experimental model of diabetes mellitus in mice, which will go on to develop diabetic retinopathy. Alloxan injection can also induce diabetes in experimental animals [19–23]. db/db mice spontaneously develop diabetes [24, 25].

### 2.1. STZ- or Alloxan-Induced Diabetic Retinopathy

Muriach et al. reported the effects of lutein on diabetic retinal changes in alloxan-induced diabetic mice [20]. These mice showed an increase of malondialdehyde (MDA, a marker for lipid peroxidation) and nuclear factor *κ*B (NF*κ*B) levels, along with a decrease in glutathione (GSH) levels and glutathione peroxidase (GPx) activities in the retina. Electroretinography (ERG) b-wave amplitude decreased in diabetic mice. Lutein [70% purity, 0.2 mg/kg body weight (wt), administered by stomach tube] was used as daily treatment started on day 4 after alloxan injection and lasted until the end of the experiment. While lutein treatment did not alter the hyperglycemic status of alloxan diabetic mice, supplementation of lutein restored levels/activities of NF*κ*B, MDA, GSH, and GPx in the retina. ERG b-wave amplitude was also restored to normal after lutein treatment, suggesting that although the high blood glucose levels were not normalized, biochemical and functional changes in the diabetic retina were improved by supplementation of lutein [20].

Sasaki et al. studied the effects of lutein on diabetic retinopathy which developed in STZ-induced diabetic mice [21]. Extracellular signal-regulated kinase signal pathway was activated in diabetic retina. Brain-derived neurotrophic factor was depleted. ERG showed a decrease of oscillatory potentials, which reflected degeneration of neurons in inner retina. At the later stage, the thicknesses of the inner plexiform layer and inner nuclear layer (INL) were decreased; the numbers of retinal ganglion cells (RGC) and inner retinal cells were reduced, together with the appearance of apoptotic cells. Reactive oxygen species (ROS) levels in the retina were also increased, which may play a role in the development of degenerative changes in the retina [21]. Mice were constantly fed either a lutein-supplemented diet [0.1% of lutein (wt/wt) added to the mouse chow] or a control diet (the same chow without addition of lutein) from the onset of diabetes until the end of the experiment. Lutein again did not affect the body weight and blood glucose levels. However, ROS levels in the retina were reduced and all diabetic pathologic changes in the retina could be avoided by the supplementation of lutein, suggesting that lutein protects the retina against diabetic damage mainly through its antioxidant effect [21]. Lutein may have potential therapeutic value in protecting visual function in patients with diabetes [19, 21].

The protective effects of zeaxanthin against diabetic retinal changes have been studied in STZ-induced diabetes in rats by Kowluru et al. [22]. Rats received powdered diet with or without supplementation of 0.02% or 0.1% zeaxanthin (equal to 8.4 and 44 mg/d) soon after induction of diabetes. The zeaxanthin levels in the retinas of normal and diabetic rats were in the range of 130 to 180 pg/mg protein and were elevated to approximately 300 and 1,500 pg/mg protein in diabetic rats receiving 0.02% and 0.1% zeaxanthin, respectively. Zeaxanthin also did not lower the high blood glucose levels in diabetic rats. In diabetic rats, the retinal levels of lipid peroxide, oxidatively modified DNA, nitrotyrosine (a parameter of oxidative stress), inducible NO synthase (iNOS), vascular endothelial cell growth factor (VEGF), and intercellular adhesion molecule- (ICAM-) 1 were all significantly increased; the expression of electron transport complex III was decreased [22]. Supplementation of zeaxanthin significantly decreased elevation of lipid peroxide, oxidatively modified DNA, nitrotyrosine, iNOS, VEGF, and ICAM-1; and the levels of electron transport complex III were increased to normal [22]. The nature defense system against oxidative stress, Mn superoxide dismutase (SOD), and GSH were also decreased significantly in the diabetics retinas [22]. Zeaxanthin supplementation increased retinal MnSOD levels to normal but the GSH levels were not completely recovered [22]. The effects of zeaxanthin were comparable in the two groups supplemented with different dosages of zeaxanthin. This study suggested that zeaxanthin has the potential to inhibit the development of diabetic retinopathy via ameliorating oxidative stress and inhibition of VEGF expression and inflammation, which raises the possibility that it could be used as an adjunct therapy to help prevent vision loss in patients with diabetes.

Effects of zeaxanthin, lutein, and other nutrients on the development of diabetic retinopathy in STZ-induced diabetic rats were recently reported by Kowluru et al. [23]. Following induction of diabetes in male rats by injection of STZ, ERG showed decreasing of the amplitudes of both a- and b-waves. Apoptotic cells increased in the retinal vasculature and degenerative capillaries could be found in the retina. Diabetes in rats caused a significant activation of retinal NF*κ*B with increase of VEGF and interleukin- (IL-) 1*β* levels in the retina [23]. Supplementation with zeaxanthin (2 mg/d), lutein (1 mg/d), lipoic acid, omega-3 fatty acids, and other nutrients ameliorated diabetes-induced capillary cell apoptosis, prevented ERG changes and activated NF*κ*B, and ameliorated increased levels of VEGF and IL-1*β* [23]. However, the severity of hyperglycemia in diabetic rats was not decreased by the supplementation, suggesting that the beneficial effects of these antioxidants on diabetes-induced retinal pathology are not due to control of hyperglycemia [23].

### 2.2. Diabetic Retinopathy in Spontaneous Diabetic Mice (db/db Mice)

In an experimental diabetes type 2 model, diabetes occurs spontaneously in the db/db mice [24, 25]. At the early stage of diabetes, retinal blood microvessels are still intact, but hyperglycemia-induced cellular oxidative stress occurs, which causes mitochondrial dysfunction, endoplasmic reticulum stress, thinning of INL and photoreceptor layers, apoptosis of RGC cells, and loss of retinal pigment epithelium (RPE) layer integrity [24]. Supplementation of wolfberry, a Chinese traditional medication containing high levels of zeaxanthin (1.76 mg/gm fruit) and lutein (0.05 mg/gm fruit), prevented retinal damage in diabetic mice. Zeaxanthin and lutein were able to mimic wolfberry's protective effect in cultured RPE cells, suggesting that they were the active agents in wolfberry's protection retinal cells against a high glucose challenge [24].

The effects of wolfberry on diabetic eye changes were investigated in a diabetic mouse model (db/db mice) by Yu et al. [25]. Wolfberry did not lower the fasting blood glucose level in db/db mice. However, in diabetic mice which showed lower levels of lutein and zeaxanthin in the retina compared with normal controls, wolfberry treatment significantly elevated zeaxanthin and lutein levels in both the retina and liver [25]. Lowered expressions of retinal scavenger receptor class B type I, glutathione S-transferase Pi, and *β*,*β*-carotene 9′,10′-oxygenase proteins were also observed in db/db mice; wolfberry elevated these protein levels back to normal, suggesting that diabetes might cause inhibition of uptake, binding and transport, and degradation of lutein and zeaxanthin within the retinal cells [25].

Hypoxia and angiogenic factors such as hypoxia-inducible factor-1*α* and VEGF and mitochondrial stress biomarker were significantly increased in the retinas of diabetic mice [25]. Wolfberry reversed these changes and increased mitochondrial biogenesis. Wolfberry also reversed mitochondrial dispersion in the RPE, increased mitochondrial copy number, and elevated citrate synthase activity [25]. All of these findings suggest that, in diabetic mice, hyperglycemia and subsequent hypoxia were causative factors leading to changes in lutein and zeaxanthin metabolic homeostasis via inhibition of metabolic gene expression producing mitochondrial dysfunction and subsequent diabetic retinal pathology [25].

## 3. Diabetic Cataract

Diabetic cataract is a common eye complication of diabetes. The effects of lutein on the prevention of diabetic cataract have been studied in a rat model [26]. Persistent hyperglycemia, high glycated hemoglobin, and loss of body weights were observed in rats treated with STZ [26]. Supplement of lutein (0.5 mg/kg orally) did not significantly affect blood glucose, glycated hemoglobin, or body weights in diabetic rats. Diabetic rats developed lens opacity in 81% (13/16) of eyes and mature cataract occurred in 7/16 eyes. In diabetic rats treated with lutein, lens opacity only developed in 38% (6/16) eyes and no mature cataracts were observed [26]. The lens in diabetic rats showed an increase of MDA levels and a decrease of GSH levels. Lutein treatment reduced the MDA levels but did not raise GSH, indicating that lutein may prevent the development of diabetic cataract through the inhibition of lipid peroxidation [26]. This study suggested that lutein might be used as an adjuvant treatment combined with the proper glycemic control to prevent the occurrence of diabetic cataract [26].

## 4. Experimental Animal Models Mimicking AMD

AMD is a major cause for irreversible blindness among the elderly in the Western world. The effects of lutein/zeaxanthin on AMD have been studied in several experimental animal models that mimic pathological changes in AMD [28–32]. The effects of lutein and zeaxanthin deficiency on the RPE and macula have been studied in nonhuman primates [33–35].

### 4.1. Apolipoprotein E-Deficient Mice

AMD is a multifactorial disease. Abnormal lipid levels and oxidative stress may contribute to the development of AMD [28, 29]. Apolipoprotein E-deficient mice (apoE−/−) are a well-established experimental animal model of hypercholesterolemia and display morphological and ultrastructural alterations in RPE similar to those in human AMD.

The effects of zeaxanthin on ocular changes in (apoE−/−) mice have been studied [29]. ApoE(−/−) mice showed elevation of plasma total cholesterol and triglycerides level. In the apoE−/− mice eyes, vacuoles in the RPE, basal laminar deposits, and an increase in Bruch's membrane thickness could be observed associated with an elevation of VEGF levels in the retina [29]. Supplementation with zeaxanthin (4 g/kg of diet) and other antioxidants (vitamin C, vitamin E, and zinc) significantly increased the retinal and liver zeaxanthin levels, decreased the VEGF levels in the retina-choroid, and improved the status of Bruch's membrane but did not change plasma cholesterol levels [29].

The effects of supplementation of lutein alone (0.093 mg/kg/d by gastroesophageal cannula) or lutein with multivitamins and GSH (vitamin A, vitamin C, vitamin E, various vitamin B compounds, *β*-carotene, GSH, and various minerals, such as zinc and selenium) on the ocular changes in apoE−/− mice were tested by the same groups of authors [28]. ApoE−/− mice showed higher plasma and retinal lipid peroxidation, with increased VEGF expression and matrix metalloproteinase- (MMP-) 2 activity in the retina-choroid, associated with ultrastructural alterations similar to AMD, such as basal laminar deposits and vacuoles, and an increase in Bruch's membrane thickness, but without drusen or neovascularization [28]. Supplements of lutein alone only partially prevented the retinal morphological changes [28]. Lutein alone caused decrease of expression of VEGF and MMP-2 in the retina-choroid; however, the difference between this group and the controls was not statistically significant [28]. Supplementation of lutein with large dose of multivitamin substantially ameliorated all retinal morphological alterations and significantly reduced VEGF levels and MMP-2 activity [28]. These results suggest that ocular changes in apoE−/− mouse could be prevented by efficient antioxidant treatments including lutein, multivitamins, and GSH [28].

### 4.2. DKO Mouse Model

The DKO mouse model is generated by knocking out a chemokine (monocyte chemoattractant protein-1 (MCP-1)) and a chemokine receptor (Cx3cr1) on a Crb1rd8 background. DKO mice develop RPE pathologic changes (lipofuscin accumulation, hypertrophy, and hypotrophy) and photoreceptor and synaptic degeneration that mimic the pathologic changes in AMD [30].

Ramkumar et al. used the DKO model to investigate the effects of lutein (1.76 *μ*M), zeaxanthin (35.1 *μ*M), long-chain n3 polyunsaturated fatty acid, docosahexaenoic acid, and eicosapentaenoic acid, which is similar to the diet used in the Age-Related Eye Diseases Study 2 (AREDS2) clinical trial [30]. DKO mice developed progressive focal photoreceptor (PR) loss and RPE mottling, whereas the controls (Crb1rd8 mice) did not develop any retinal lesions [30]. DKO mice developed accumulation of liposomes and lipofuscin in the RPE, focal RPE hypertrophy and hypopigmentation, loss of the outer and inner segments layers, and abundant PR loss [30]. AREDS2-treated DKO mice showed a significantly more healthy RPE and PR [30]. DKO mice had high A2E levels and mRNA levels of inflammatory genes [*tumor necrosis factor-α, cyclooxygenase-2* (*COX-2), IL-1β, and iNOS*] and angiogenic genes (*VEGF*), which were significantly greater than those of AREDS2-treated DKO mice, indicating that AREDS2 could downregulate the expression of inflammatory and proangiogenic genes linked to advance AMD [30]. This study demonstrated a benefit of the AREDS2 diet on retinal AMD-like lesions in DKO model [30].

### 4.3. Mice Lacking *α*v*β*5 Integrin

Mice lacking *α*v*β*5 integrin (*β*5−/− mice) have a primary defect in phagocytic activity due to lack of the outer segment recognition receptor *α*v*β*5 integrin; this causes accelerated age-accumulation of lipofuscin, increases in 4-hydroxynonenal-adducts, and decreased solubility of actin in the RPE, accompanied by a decrease of a-wave amplitude in ERG that indicates dysfunction of photoreceptors [31]. Accumulation of lipofuscin, damage to the RPE and PR in aged *β*5−/− mice, mimics the pathologic changes of dry-type AMD in human [31]. Supplementation of grapes or marigold extract containing lutein/zeaxanthin (52 mg lutein/2 mg zeaxanthin per kg body wt/d) given to *β*5−/− mice prevented 4-hydroxynonenal-adduct formation and decreased actin solubility and lipofuscin accumulation and age-related photoreceptor dysfunction. This suggests that consumption of an antioxidant-rich diet might prevent the destabilization of the actin resulting from a physiological, sublethal oxidative burden on RPE cells, which appears to be associated with age-related blindness [31].

### 4.4. LASER-Induced Choroidal Neovascularization

Choroidal neovascularization (CNV) is an important pathological change in the neovascular AMD. CNV can be induced in mice by LASER photocoagulation. Izumi-Nagai et al. reported that pretreatment with oral supplements of lutein (10–100 mg/kg body wt per day) significantly suppressed the index of CNV volume induced by LASER photocoagulation [32]. Macrophage infiltration into CNV is decreased in lutein-treated mice. LASER-induced elevation of ICAM-1, VEGF, and MCP-1 levels in RPE-choroid was also significantly inhibited by lutein treatment [32]. LASER-induced CNV was accomplished with NF*κ*B activation, which was also prevented by supplementation of lutein [32]. This study suggested that lutein suppressed LASER photocoagulation-induced CNV through inhibition of NF*κ*B activation which upregulates inflammatory molecules and angiogenic factors, suggesting that lutein supplementation may have therapeutic value in suppressing the development of CNV [32].

### 4.5. Lutein and Zeaxanthin Deficiency Studies in Monkeys

Effects of lutein and zeaxanthin on the RPE have also been studied in nonhuman primates [33–35]. Comparison of monkeys on a standard diet or xanthophyll free diet demonstrated that deficiency of lutein and zeaxanthin leads to an absence of macular pigment, increased hyperfluorescence in the foveal region [33], and decreased RPE density at the foveal center [34], suggesting that the RPE cells are sensitive to the absence of macular pigment. In monkeys fed a normal diet with normal macular pigmentation, the fovea was less sensitive to blue-light-induced damage than the parafovea [35]. In xanthophyll-free monkeys, the fovea sensitivity to blue-light-induced damage was increased to the same level as that of parafovea. Lutein and zeaxanthin supplementation decreased foveal sensitivity to normal, indicating that lutein and zeaxanthin provide significant protection against short-wavelength photochemical damage in the fovea [35]. This study suggested that supplementation of lutein and zeaxanthin might contribute to the reduction of risk for AMD, especially for persons with reduced macular xanthophyll levels due to retinal disease, poor diet, or genetic predisposition [35].

## 5. Retinal Ischemic/Hypoxic Injury

Effects of lutein on retinal ischemic/hypoxic injury have been studied in two different experiment animal models: blockade of internal carotid artery-induced ischemia model in mice [36–38] and high intraocular pressure- (IOP-) induced ischemia model in rats [39, 40].

### 5.1. Retinal Ischemic/Hypoxic Injury Induced by Blockade of Internal Carotid Artery

Retinal ischemia is a common feature of retinal vasculopathies, such as retinopathy of prematurity and diabetic retinopathy. In ischemic retinopathy, decrease in the normal retinal blood supply results in oxidative stress and retinal neovascularization [36].

Retinal ischemia/reperfusion (I/R) was induced by a blockade of internal carotid artery in mice [37, 38]. Lutein (0.2 mg/kg) or vehicle (DMSO) was given by intraperitoneal injection 1 h before and after reperfusion. In the ischemic group, Muller cell gliosis was induced by retinal I/R injury and combined with a marked cell loss and appearance of apoptotic cells in the ganglion cell layer (GCL) and the INL, suggesting the involvement of RGC and amacrine cells. Bipolar and horizontal cells seemed relatively unaffected by I/R [37, 38]. In flash ERG, the b-wave/a-wave ratio and oscillatory potentials were significantly reduced [38]. Oxidative stress and oxidative DNA damage were increased in GCL and INL layers [38]. Lutein treatment prevented the deterioration of ERG, decreased the oxidative stress, decreased the cell loss and the apoptotic cells in the retina, inhibited upregulation of glial fibrillary acidic protein, which avoided Muller cell gliosis, and preserved retinal function [37, 38]. These studies suggested that lutein protected the retina, especially the RGC, from I/R damage by its antioxidative, antiapoptotic, and anti-inflammatory properties [37, 38].

### 5.2. High Intraocular Pressure Induced-Ischemia Model

It is well known that I/R generates retinal damage, particularly if ischemia persists for ≥60 min [39]. Most of the retinal damage is caused during reperfusion rather than during ischemia [40, 41]. ROS and reactive nitrogen species play important roles in the pathogenesis of I/R induced retinal damage [40].

The effect of lutein on high IOP-induced retinal ischemic/reperfusion damage has been tested in a rat model [40] by increasing IOP above systolic blood pressure for one hour in Sprague-Dawley rats. Lutein was injected intravitreally (10 *μ*L of 0.5 mg/mL) 30 minutes before ischemia or intraperitoneally (0.5 mg/kg) one hour before and one hour after ischemia. The experimental rats were sacrificed 24 hours after reperfusion. Acute ischemic injury caused loss of 46% and 32% cells in the GCL and INL, respectively [40]. Lutein injected intraperitoneally (0.5 mk/kg of body weight) significantly increased survival of retinal neurons to 85% and 88% in the GCL and INL, respectively. Neuronal nitric oxide synthase (nNOS) was increased in early ischemic retina and this can induce excessive nitric oxide (NO), which results in neuronal cell death and activation of glial cells [40]. nNOS and COX-2 (related to prostaglandin metabolism) were increased in ischemic retinas and these increases were inhibited by lutein [40]. This study confirmed that lutein functions as an anti-ischemic drug by inhibiting nNOS and COX-2 expressions and suggested that lutein supplementation may protect against ischemia-mediated cell death in the retina [40].

In another study in high IOP-induced ischemic retina model in rats, the effects of subcutaneous injections of lutein (100 mg/kg/d) have been compared to three other antioxidants (vitamin E and two extracts from plants: Trigonella and Teucrium) [39]. I/R caused severe retinal damage as indicated by increased levels of MDA and caspase-3 activity (biomarker of apoptosis) and decreased GSH contents. Lutein significantly prevented all deteriorative changes caused by I/P and showed the strongest effect of the antioxidants tested, whereas other antioxidants only had partially protective effects [39]. This study suggested that lutein showed superior efficacy compared to all other tested antioxidants. Therefore, the future therapeutic use of lutein against I/P damage of the retina deserves further experiments and clinical trials [39].

## 6. Light-Induced Retinal Damage

The effects of zeaxanthin on light-induced photoreceptor cell death have been studied in quail fed a carotenoid-deficient diet [42]. Light irradiated retina showed numerous apoptotic PR with apoptotic rods outnumbering cones, accompanied by activated microglial invasion of the damaged PR layer. Oral supplementation of zeaxanthin produced a rapid enrichment in the zeaxanthin fraction of total serum xanthophylls and a slow increase in the zeaxanthin fraction of retinal xanthophylls. Zeaxanthin supplementation decreased apoptosis and activation of microglia. The numbers of apoptotic PR and activated microglia correlated negatively and significantly with the concentration of retinal zeaxanthin, suggesting that zeaxanthin could protect PR against light-induced apoptosis [42].

The effects of lutein in light-induced retinal degeneration have been tested in a mouse model of light-induced retinal degeneration [43]. In this model, after the light irradiation, PR cells gradually degenerate and become apoptotic, resulting in thinning of the PR layer. ERG shows a reduction of a-wave and b-wave amplitudes, indicating visual impairment. Apoptosis is caused by upregulation of double-stranded breaks in DNA [43]. Supplementation of lutein in powder chow (170 mg/kg body wt/d) attenuated apoptosis of PR by suppression of double-stranded breaks in DNA. Light-induced oxidative stress of the retina and functional and histological damage in the retina were suppressed by the treatment of lutein. This study indicated that a lutein-supplemented diet could attenuate light-induced visual impairment by protecting the DNA of the PR cells [43].

## 7. Rescue of PR in Retinal Detachment

The neuroprotective effect of lutein on the PR in a rat model of retinal detachment has been studied [44]. Retinal detachment was induced by subretinal injections of sodium hyaluronate in rats. Daily injections of corn oil (control group) or lutein (0.5 mg/kg) in corn oil (treatment group) were administered intraperitoneally. In the control group, the average number of viable cells in the outer nuclear layer (ONL) was significantly decreased. Apoptotic cells could be detected in the cells in the ONL. Lutein treatment, commenced after four hours, significantly increased the number of viable cells and decreased the number of apoptotic cells. Cell loss in the ONL was reduced from 24% in the control group to 11% in lutein treatment group, demonstrating that lutein could prevent 54% of cell death caused by retinal detachment. Western blotting results showed that there was a decrease of cleaved caspase-8 and caspase-3, but not caspase-9, in lutein treated group, indicating that lutein probably acted on the extrinsic apoptosis pathway rather than the intrinsic apoptosis pathway [44]. Lutein treatment group also showed significantly reduced glial fibrillary acidic protein immunoreactivity and preserved rhodopsin expression. Similar results were detected when lutein was given 36 hours after retinal detachment induction [44]. This study suggests that lutein is a neuroprotective agent that could rescue the apoptosis of PR in rats with retinal detachment even when given 36 hours after the occurrence of retinal detachment [44]. The use of lutein in retinal detachment patients may serve as an adjunct to surgery for the improvement of visual outcomes [44].

## 8. Retinitis Pigmentosa

Retinitis pigmentosa is a group of inherited disorders characterized by progressive PR degeneration leading to loss of vision. The rd1/rd1 mouse has an insertion of viral DNA in the *β*-subunit of the* cGMP phosphodiesterase* gene. This mutation leads to toxic accumulation of cGMP and high Ca2+ levels in the rd1 photoreceptors and causes the death of rods followed by cone death [45–47]. A mutation in the same gene has been found in human forms of autosomal recessive retinitis pigmentosa, making the rd1 mouse an ideal spontaneous model of retinitis pigmentosa [45]. It has been reported that cone cell death after rod cell death in the rd1 mouse model was due to oxidative damage caused by reduced oxygen utilization by rods and that antioxidant therapy might prevent the cone cell death in this model [45–47].

The effects of oral administrated antioxidant mixture which contained zeaxanthin and lutein, *α*-lipoic acid, and GSH plus an extract obtained from* Lycium barbarum* that contains various antioxidants, such as zeaxanthin and *β*-carotene, have been tested in rd1 mouse [45]. Apoptotic cells could be detected in the ONL of rd1 mice retina by terminal deoxynucleotidyl transferase dUTP nick end labeling assay. Treatment with lutein or zeaxanthin alone decreased apoptotic cells in this model by 11% and 20%, respectively, which was not statistically significant [45]. But treatment with the full antioxidant mixture resulted in a statistically significant decrease (39%) of apoptotic cells [45, 47]. Cells with oxidative DNA damage could be detected in the ONL of rd1 mice retina by avidin staining. Treatment with lutein or zeaxanthin alone decreased avidin-positive cells by 14%, which was also not statistically significant. Only the full antioxidants mixture reduced the avidin-positive cells by a statistically significant 21% [45, 47]. Supplement of antioxidants mixture also increased GPx activity and GSH levels and decreased cystine levels in rd1 retinas, whereas no change was observed in glutathione disulfide reductase activity [45, 47]. These studies suggest that oxidative stress plays a role in the photoreceptor death in retinitis pigmentosa and an antioxidant mixture containing zeaxanthin and lutein is able to reduce photoreceptor death in rd1 retina [45, 47].

## 9. Endotoxin-Induced Uveitis

Intraperitoneal or subcutaneous injection of lipopolysaccharide (LPS) into mice or rats can generate endotoxin-induced uveitis (EIU), which is an important experimental animal uveitis model [19, 48–51, 53, 54].

Intravenous injection of lutein suppresses the development of EIU in rats in a dose-dependent manner [48]. The anti-inflammatory effect of lutein (100 mg/kg) is comparable to dexamethasone (1 mg/kg). During the development of EIU, various inflammatory factors significantly increased in the aqueous humor, including tumor necrosis factor-*α*, IL-6, MCP-1, macrophage inflammatory protein-2, prostaglandin E2, and NO. Lutein significantly decreased the levels of these inflammatory factors in the aqueous humor [48]. The mechanism of the anti-inflammatory effect of lutein may be related to the suppression of proinflammation signal pathways. Lutein inhibited the activation of NF*κ*B in the iris-ciliary body and the expression of iNOS and COX-2 in cultured mouse macrophage cells, suggesting that lutein can have a potent anti-inflammatory effect on EIU [48].

Another study of the effects of lutein in EIU showed that levels of NO and MDA were increased in ocular tissues in EIU model [49]. The levels of oxygen radical absorbance capacity, GSH, vitamin C, total superoxide dismutase SOD, and GPx activities and mRNA levels of* copper-zinc SOD*,* manganese SOD*, and* GPx* were reduced in ocular tissues in EIU [49]. Supplementation of lutein in drinking water (0.125–0.25 mg/kg/d) for five days before LPS injection significantly avoided the above-mentioned changes related to oxidative stress. These results suggested that the protective effects of lutein against the LPS-induced inflammation might be related to the alleviation of oxidative stress [49].

In EIU, inflammation processes involve both uvea and retina. Inflammation in the retina causes dysfunction of retina and loss of vision. The outer segments of PR are shortened, accompanied by a reduction in rhodopsin and a decrease of photoreceptor cell function. ERG shows a decrease in the a-wave amplitude [19, 50]. Sasaki et al. reported that lutein had a neuroprotective effect during retinal inflammation in EIU [50]. Supplementation of lutein by subcutaneous injection (100 mg/kg body wt) prevented the shortening of the outer segments and the reduction of rhodopsin. ERG a-wave amplitude was preserved by the treatment of lutein. Pathological changes of Muller cells and activation of signal transducer and activator of transcription 3 were also decreased by lutein. This study indicated that lutein protected the neuroretina against inflammatory damage by reducing oxidative stress, suppressing inflammatory signal pathway, and preservation of rhodopsin protein [19, 50].

## 10. Miscellaneous

The effects of zeaxanthin on the retinal antioxidative capacity, the levels of lipid and protein peroxidation, and their signal pathway were tested in normal rats [51]. Rats fed with high dosages of zeaxanthin showed a significant increase of Akt phosphorylation in the retina that might activate the NF-E2-related factor 2 (Nrf2) pathway. GSH levels and the expression of Nrf2 target genes in the retina were significantly increased by zeaxanthin supplementation [51]. The biomarker of lipid and protein peroxidation (4-hydroxynonenal and the carbonyl protein) were significantly decreased in the retina of zeaxanthin-supplemented rats [51]. Rats fed with a lower dosage of zeaxanthin showed a tendency toward similar changes as the high dosage zeaxanthin group but statistical significance was reached in only some of the parameters [51]. The authors mentioned that this is the first time that zeaxanthin was presented as a phase II enzymes inducer, instead of an antioxidant [51].

## 11. Summary

The effects of lutein and zeaxanthin on the prevention of various eye diseases, including AMD, diabetic retinopathy and cataract, ischemic/hypoxia induced retinopathy, light damage of the retina, retinitis pigmentosa, retinal detachment, and uveitis [19–51], have been studied in a variety of experimental animal models. In these animal models, lutein and zeaxanthin have been reported to have beneficial effects in protecting ocular tissues and cells, especially the retinal neurons, against damage caused by different etiological factors [19–51]. The mechanisms responsible for these effects of lutein and zeaxanthin include prevention of phototoxic damage by absorption of blue light [53], reduction of oxidative stress through antioxidant activity and free radical scavenging [54], and their anti-inflammatory and antiangiogenic properties [4].

The therapeutic effects of lutein and zeaxanthin have been evaluated clinically in several eye diseases such as AMD, diabetic retinopathy, retinopathy of prematurity, and cataract [1, 3, 10, 23] and also in a small group of retinitis pigmentosa patients [3]. Lutein and zeaxanthin have not been tested clinically in other eye diseases, such as retinal detachment (for neuroprotective effects on the photoreceptors) and uveitis (anti-inflammation and improvement of survival of retinal neurons). Future studies to explore the effects of supplementation of lutein and zeaxanthin in these severe eye diseases may prove to be clinically valuable.

Experimental animal studies have provided important information on the effects of lutein and zeaxanthin in various eye diseases. However, animal models also have their limitations and many problems should be addressed prior to clinical application.

The first limitation involves species differences between human and experimental animals. Most of these studies on the effects of lutein and zeaxanthin were tested in the murine models. Rats and mice have the advantage that they are relatively inexpensive, easily performed, and reproduced. However, rodents have no macula and their retinal photoreceptor cells are predominantly rods, rather than the cone cells that predominate in the human macula. These differences should be considered in the translation of results obtained from animal studies to human eye diseases, especially in AMD [10].

Second, the dosages used in animal studies are usually much higher than those used clinically. The tested dosages of lutein (10 mg/d) and zeaxanthin (2 mg/d) in AREDS2 are much lower than the dosages used in animal studies. Many animal studies have indicated that the effects of both lutein and zeaxanthin are dose-dependent [29, 32, 51]. The effective dosages are usually at the range of 0.5–100 mg/kg body wt/d (oral administration), which translates to 30–6000 mg/d in a 60 kg weight adult [26, 31, 32, 42, 49, 51], which is usually 60- to 300-fold greater than previously tested clinical dosages. For example, Zou et al. reported that rats fed with high dosages of zeaxanthin (24 mg/kg/d, equal to 1440 mg/d in a 60 kg weight adult) showed a significant activation of the Nrf2 pathway in rats [51]. A lower dosage (8 mg/kg/d, equal to 480 mg/d in a 60 kg weight adult) showed similar tendency but the difference between treated rats and the controls was not statistically significant [51]. Therefore, it may be necessary to increase clinical trial dosages of lutein and zeaxanthin in order to reproduce the results obtained in animal studies. This will require study of the safety and toxicity of lutein and zeaxanthin at large dosages to determine maximum dosages that could be used clinically safely.

Third, there are currently three known macular pigments, lutein, zeaxanthin, and meso-zeaxanthin [52]. In the past, most animal studies tested only the effects of lutein and zeaxanthin. Very little is known on the function of meso-zeaxanthin [55–57]. Therefore, future studies will need to include the effects of meso-zeaxanthin in various animal models to elucidate the full picture of the role of macular pigment in ocular disease.

## Figures and Tables

**Table 1:** Effects of lutein and zeaxanthin on experimental animal models for ocular diseases.

	Authors	Year	Animal	Methods for inducing disease	Pathologic changes	Xanthophylls used	Effectiveness
Diabetic retinopathy	Muriach et al. [20]	2006	Mice	Diabetes Alloxan-induced	NF*κ*B, ROS & MDA (+) ERG damage Antioxidant (−)	L (oral)	Prevent all changes
	Sasaki et al. [21]	2010	Mice	Diabetes STZ-induced	ROS & extracellular signal-regulated kinase (+) Brain-derived neurotrophic factor (−) ERG damage RGC apoptosis	L (oral)	Prevent all changes
	Kowluru et al. [22]	2008	Rats	Diabetes STZ-induced	ROS, VEGF, ICAM & reactive nitrogen species (+) Antioxidant (−)	Z (oral)	Prevent all changes
	Kowluru et al. [23]	2014	Rats	Diabetes STZ-induced	ERG damage NF*κ*B, VEGF & IL-1*β* (+) Cell apoptosis	Z, L, and others (oral)	Prevent all changes
	Tang et al. [24]	2011	Mice	db/db mice	RPE & RGC damage Mitochondria dysfunction	Wolfberry (L Z) (oral)	Prevent all changes
	Yu et al. [25]	2013	Mice	db/db mice	VEGF (+) antioxidant (−) Mitochondria dysfunction	Wolfberry (L Z) (oral)	Prevent all changes

Diabetic cataract	Arnal et al. [26]	2009	Rats	Diabetes STZ-induced	Lens opacity MDA (+) GSH (−)	L (oral)	Prevent all changes except GSH

Age-related macular degeneration	Fernández-Robredo et al. [29]	2009	Mice	ApoE−/− mice	RPE & Bruch's membrane changes VEGF (+)	Z & other antioxidants (oral)	Prevent all changes at high doses
	Fernández-Robredo et al. [28]	2013	Mice	ApoE−/− mice	RPE & Bruch's membrane changes MMP-2 & VEGF (+)	L & others or L alone (oral)	Only effective in L with others
	Ramkumar et al. [30]	2013	Mice	DKO mice	RPE & PR damage COX-2, IL-1, iNOS & VEGF (+)	L, Z, and others (oral) (AREDS2)	Prevent all changes
	Yu et al. [31]	2012	Mice	*β*5−/− mice	RPE & PR damage Lipofuscin (+) Actin solubility (−)	L, Z, and others (oral)	Prevent all changes
	Izumi-Nagai et al. [32]	2007	Mice	LASER-induced CNV	CNV NF*κ*B, VEGF, ICAM-1 & MCP-1 (+)	L (oral)	Prevent all changes at high doses

Ischemic/hypoxia retinopathy	Li et al. [38]	2009	Mice	Carotid artery block	RGC & ERG damage Muller cells activation ROS (+)	L (IP)	Prevent all changes
	Li et al. [37]	2012	Mice	Carotid artery block	ERG damage ROS (+) Muller cells activation	L (IP)	Prevent all changes
	Choi et al. [40]	2006	Rats	High IOP	Cell death nNOS & COX-2 (+)	L (IP or IVt)	Prevent all changes
	Dilsiz et al. [39]	2006	Rats	High IOP	MDA & caspase-3 (+) GSH (−)	L (IC)	Prevent all changes

Light damage retinopathy	Thomson et al. [42]	2002	Quails	Intensive white light	PR apoptosis	Z & L (oral)	Prevent all changes
	Sasaki et al. [43]	2012	Mice	Intensive white light	PR apoptosis & DNA damage, ERG damage	L (oral)	Prevent all changes

Retinitis pigmentosa	Miranda et al. [45]	2010	Mice	rd1 mice	PR apoptosis GSH & GPx (−) MDA (+)	L, Z & other antioxidants or L & Z alone (oral)	Effective only in L, Z & other antioxidants

Retinal detachment	Woo et al. [44]	2013	Rats	Hyaluronate subretinal injection	PR apoptosis Caspase-8 & caspase-3 (+) Glial cell activation	L (IP)	Prevent all changes

Uveitis	Jin et al. [48]	2006	Rats	LPS (SCI)	Proinflammatory factors, NF*κ*B & iNOS (+)	L (IV)	Prevent all changes
	He et al. [49]	2011	Mice	LPS footpad injection	NO & MDA (+) SOD & GPX (−)	L (oral)	Prevent all changes
	Sasaki et al. [50]	2009	Mice	LPS (IP)	PR & ERG damage ROS, IL-6, STAT-3 (+)	L (IC)	Prevent all changes

Misc.	Zou et al. [51]	2014	Rats	Normal	—	Z (oral)	Akt, Nrf2 & GSH (+) Peroxidation (−)

L: lutein; Z: zeaxanthin; IC: subcutaneous injection; IP: intraperitoneal injection; IV: intravenous injection; IVt: intravitreous injection. (−): decreased; (+): increased.

For other abbreviations, see the text.
